# Changes in Alcohol Consumption during the COVID-19 Pandemic—Small Change in Total Consumption, but Increase in Proportion of Heavy Drinkers

**DOI:** 10.3390/ijerph18084231

**Published:** 2021-04-16

**Authors:** Ingeborg Rossow, Elin K. Bye, Inger Synnøve Moan, Carolin Kilian, Jørgen G. Bramness

**Affiliations:** 1 Department of Alcohol, Tobacco and Drugs, Norwegian Institute of Public Health, 0213 Oslo, Norway; ElinKristin.Bye@fhi.no (E.K.B.); IngerSynnove.Moan@fhi.no (I.S.M.); JorgenGustav.Bramness@fhi.no (J.G.B.); 2 Institute of Clinical Psychology and Psychotherapy, Technische Universität Dresden, 01187 Dresden, Germany; carolin.kilian@mailbox.org; 3 Institute of Clinical Medicine, UiT—The Arctic University of Norway, 9019 Tromsø, Norway; 4 Norwegian National Advisory Unit on Concurrent Substance Abuse and Mental Health Disorders, Innlandet Hospital Trust, 2381 Brumunddal, Norway

**Keywords:** alcohol use, changes, COVID-19, Norway, heavy drinkers, distribution of consumption

## Abstract

Little is known about possible changes in alcohol consumption distribution during the COVID-19 pandemic. We estimated how individual changes in alcohol consumption during the pandemic translated into changes in: (i) mean consumption; (ii) dispersion of consumption distribution; and (iii) prevalence of heavy drinkers. We employed data from two independent web-surveys of Norwegian adults collected between April and July 2020 and limited to those reporting past year alcohol consumption (*N*_1_ = 15,267, *N*_2_ = 1195). Self-reports of changes in drinking behavior were quantified, assuming change being relative to baseline consumption level. During the pandemic, we found a small increase (Survey 1) or no change (Survey 2) in estimated mean alcohol consumption (which parallels to total consumption). However, in both surveys, the dispersion of the distribution increased significantly (*p* < 0.001). For most respondents, an average modest decline in consumption was found. However, the small fraction with the highest baseline consumption increased their consumption substantially, and in effect, the proportion of heavy drinkers increased markedly (*p* < 0.001). In conclusion, quantifications of reported changes in alcohol consumption during the pandemic suggest that the upper 5 to 10% of the drinkers increased their consumption and hence the prevalence of heavy drinkers increased, despite little or no change in total alcohol consumption.

## 1. Introduction

Alcohol consumption is among the leading risk factors for premature death and loss of healthy life years globally [1]. With increasing consumption, the individual risk of injuries or disease increases [2]. In addition, at the population level, changes in total alcohol consumption are accompanied by changes in prevalence of heavy drinking [3,4] (i.e., drinking above a certain cut-off, for example, more than 14 drinks per week [5]). Changes in total alcohol consumption are also accompanied by changes in rates of alcohol-related harms [6], and thus, an increase in consumption implies an increase in the population prevalence of heavy drinking and incidence of alcohol-related harms, and vice versa. This important public health effect of the overall alcohol consumption level in a society, reflects a consistent pattern of regularity in the distribution of alcohol consumption across various populations and societies; it is highly skewed, and there is a strong association between the arithmetic mean and the dispersion of alcohol consumption [6,7,8]. One implication of this regularity is a strong association between the mean alcohol consumption per drinker in a population (which corresponds to total consumption) and the prevalence of heavy or excessive drinking or alcohol-related harms [6,8]. Implicitly, this leads us to the expectation that when the mean consumption remains stable, so does the prevalence of heavy drinking.

During the first months of the COVID-19 pandemic, many survey studies found that a large proportion of current drinkers reported they had changed their consumption, either by drinking less or by drinking more [9,10,11,12,13,14,15,16,17,18,19,20,21,22]. In most of these studies, the proportion having decreased their consumption was larger than that reporting increased consumption. This tendency for more people to report a decrease, could be interpreted as an indication of a decrease in overall consumption, which in turn would be expected due to reduced affordability and less social availability during the pandemic [23]. However, individual changes in consumption seem to be contingent on the initial drinking level; several studies found that those with initial low consumption tended to report less drinking during the pandemic, and those with a high initial level, tended to report more drinking [9,20,22,24]. These findings may suggest that despite a preponderance of people reporting reduced drinking, the overall consumption—in the survey samples—is not necessarily reduced.

Moreover, if we assume that a change in consumption is proportional to the initial consumption level, the differential changes by initial drinking level likely implied an increase in the dispersion of the distribution. Based on Weber–Fechner’s law in psychology, as applied to alcohol consumption [4], the assumption is that an increase (or decrease) in consumption is proportional to the initial consumption level. As an illustrative example, Skog argued that a person consuming 20 L per year will perceive an increase of 5 L as comparable to an increase of 1 L by a person who drinks 4 L per year [4]. Thus, in the context of the COVID-19 pandemic, it is conceivable that the reported changes in consumption in both directions during the first months of the pandemic, meant that the average consumption remained approximately the same, while the dispersion of the distribution increased, and thus also the prevalence of heavy drinkers. This issue is the focus of the present study, and to our knowledge, it has—to this end—not been examined empirically.

Against this backdrop, we explored how individual changes in alcohol consumption during the first months of the COVID-19 pandemic in Norway translated into possible changes in: (i) average consumption among adult drinkers, (ii) dispersion of consumption distribution, and (iii) prevalence of heavy drinkers.

## 2. Materials and Methods

### 2.1. Samples and Data Collections

We employed two data sets from surveys among Norwegian adults, collected quite shortly after the COVID-19 pandemic took hold in Europe. Survey 1 stems from the European Alcohol Use and COVID-19 survey (ESAC), an online survey targeted to adults aged 18 years or older which captured changes in alcohol consumption during the first months of the COVID-19 pandemic [25]. The online survey was available in 20 languages and took place in 21 European countries, including Norway. Survey translations and an outline of dissemination strategies employed are available online [26]. The Norwegian part of the survey was conducted between the end of April and June, and respondents were recruited from alcohol research and policy networks, social media, the Norwegian Institute of Public Health web page, and one online version of a large national newspaper. In this way, we obtained a convenience sample consisting of 17,092 individuals. Participation in the survey was voluntary and anonymous, and the survey was approved by the Data Protection Officers of the Technische Universität Dresden (Germany) and of the Norwegian Institute of Public Health with regards to the EU General Data Protection Regulation 2016/679.

Survey 2 was a web-survey on alcohol use, conducted by a Norwegian data collection unit (Opinion) in June–July 2020 on behalf of the Norwegian Directorate of Health (Opinion survey). Respondents were 18 years and older and randomly selected from a national web panel. The net sample comprised 1328 respondents (27.4% response rate).

### 2.2. Measures

In both surveys, respondents were asked about their alcohol consumption in the past 12 months, hereafter referred to as baseline alcohol consumption, using the AUDIT-C questionnaire [27]. For this study, we considered the first two items (frequency of drinking, quantity of alcohol consumed per occasion) (see Table S1 for response options). In the ESAC survey, standard units of alcohol were described per beverage type and corresponded to approximately 10 g (see [22] for details), whereas in the Opinion survey, an alcohol unit was described as a drink of spirits, a glass of wine or a small bottle of beer. Baseline alcohol consumption was estimated as the product of drinking frequency in the past 12 months and usual quantity per occasion (see Table S1 for recoding of variables) and divided by 52 to obtain number of units consumed per week.

In both surveys, respondents who reported past-year alcohol consumption were also asked about perceived changes in alcohol use during the pandemic, as compared to previously. In the ESAC survey, respondents were asked about perceived changes in drinking frequency and in quantity per occasion in the past month, whereas in the Opinion survey the respondents were asked whether their alcohol consumption had changed during the period with the pandemic, as compared to previously. Both surveys had the following five response options: ‘much less (often)’, ‘slightly less (often)’, ‘no change’, ‘slightly more (often)’ and ‘much more (often)’. Based on these responses, we estimated alcohol consumption during the pandemic. In line with Skog, these calculations were based on the assumption that reported changes in drinking behavior depend on initial consumption [4]. For example, the increase in quantity per occasion when reporting ‘much more’ is likely larger in absolute terms when the initial consumption is high as compared to low. We applied three different models of quantifiable relative changes (see Table S1 for detailed description). In Model 1, we assumed a small relative change from the initial level (e.g., much more = +30%), in Model 2, a medium relative change was assumed (e.g., much more = +50%), and in Model 3, a large relative change (e.g., much more = +100%). Based on these models, we calculated alcohol consumption during the pandemic. For the ESAC survey, we applied the three models to create variables for drinking frequency and usual quantity during the past month, and thereby, three variables (one for each model) for volume of consumption during the pandemic (also presented as number of units per week). For the Opinion survey, we calculated consumption during the pandemic as baseline consumption times relative change (e.g., for Model 1, those reporting to drink ‘much more’, consumption during the pandemic was 130% that of their baseline consumption). As a sensitivity test, we also estimated consumption per week under the assumption that reported changes in frequency and usual quantity were absolute, rather than relative to initial consumption (Model 4, Supplementary Table S1). The magnitude of the relative change assumed in Models 1 through 3, is likely conservative, considering the substantial individual flux in alcohol consumption from one year to the next, as reported from various countries including the Nordic countries and the USA [28].

There is no single way of operationalizing heavy drinking. Thus, we estimated the proportion of heavy drinkers from each of the consumption distribution variables, applying three different cut-offs for risk drinking; 14+ units/week, 21+ units/week and 28+ units/week, which reflect the varying limits for risk drinking in various countries [5].

### 2.3. Statistical Analyses

The analyses were explorative. The sample distribution according to gender, age and educational level in the ESAC survey deviated from that in the Norwegian adult population [29], and hence sample weights [30] were applied in the analyses. The Opinion survey sample was also weighted by gender, age and residence area (Table 1). Descriptive statistics (mean, standard deviation and percentiles) were calculated for the distributions of alcohol consumption at baseline and estimates of consumption during the pandemic. Differences in dispersions were tested with the Kolmogorov–Smirnov test. Differences in prevalence of heavy drinkers between baseline and during the pandemic were tested with Z-test. The analyses were conducted in SPSS version 26.

## 3. Results

The analytical samples comprised current drinkers with valid answers on alcohol consumption in the past 12 months and perceived changes in drinking. For the ESAC survey (9.3% abstainers, 1.4% invalid responses, *n* = 15,267) and the Opinion survey (10.0% abstainers, 0% invalid responses, *n* = 1195), the average age was 43.8 years (SD = 15.1) and 47.7 years (SD = 17.1), respectively. Gender and age category distributions of the two weighted survey samples are presented in Table 1.

For baseline alcohol consumption, estimated average per drinker was higher in the ESAC survey (5.5 units per week) than in the Opinion survey (4.0 units per week). As expected, in both surveys, the distribution was skewed with the median being much lower than the mean (Table 2).

**Figure 1:**
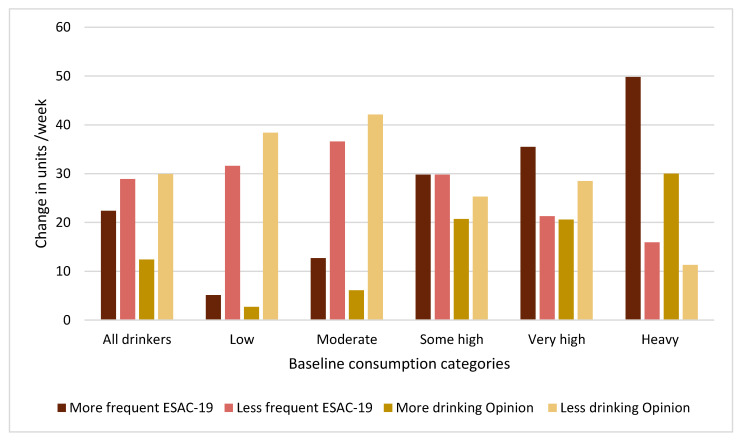
Self-reported changes in drinking behavior by baseline consumption categories and survey. Note: the baseline consumption categories are based on percentiles. Low = < 25th percentile, moderate = between the 25th and 50th percentiles, some high = between the 50th and 75th percentiles, very high = between the 75th and 90th percentiles, and heavy = > 90th percentile; more frequent = reporting ‘much more often’ or ‘slightly more often’ on change in drinking frequency in the past month; less frequent = reporting ‘much less often’ or ‘slightly less often’ on change in drinking frequency in the past month; more drinking = reporting ‘much more’ or ‘slightly more’ on change in alcohol consumption during the pandemic; less drinking = reporting ‘much less’ or ‘slightly less’ on change in alcohol consumption during the pandemic.

In the ESAC survey, almost half (48.7%) reported no change in drinking frequency, 28.9% reported they drank less often, and 22.4% reported they drank more often. A majority (60.2%) reported no change in usual quantity per occasion, whereas 26.8% reported a decrease and 13.0% reported an increase. In the Opinion survey, more than half (57.4%) reported no change in their alcohol consumption, 29.9% reported they drank less, and 12.8% reported they drank more (Figure 1). In both surveys, the extent to which respondents reported to have decreased or increased their drinking depended on their baseline consumption. Thus, the proportion reporting more drinking (i.e., more frequent drinking or a higher amount per occasion in the ESAC survey and more drinking in the Opinion survey) increased with increasing baseline consumption level (Figure 1).

Assuming that reported change in alcohol consumption was relative to baseline consumption, estimated mean consumption during the pandemic was—compared to baseline—somewhat higher in the ESAC survey and the same in the Opinion survey (Table 2). Moreover, in both surveys, the 90th and 95th percentiles were higher for the estimated consumption during the pandemic, whereas the median and 25th percentiles were mainly lower, as compared to baseline. Thus, while the change in mean consumption was small, the dispersion of the distribution increased (Table 2). The distributions for all three models assuming relative change differed significantly from the distribution of baseline consumption (*p* < 0.001).

In the sensitivity analyses, we modelled change in consumption as absolute rather than relative to baseline consumption. In Model 4, the mean consumption remained the same in both surveys, whereas the dispersion increased in the ESAC survey (*p* < 0.001) and in the Opinion survey (*p* < 0.001) (Table 2).

**Figure 2:**
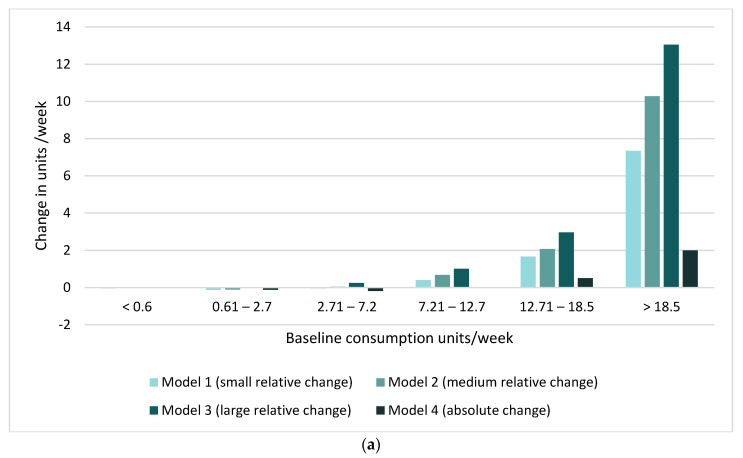

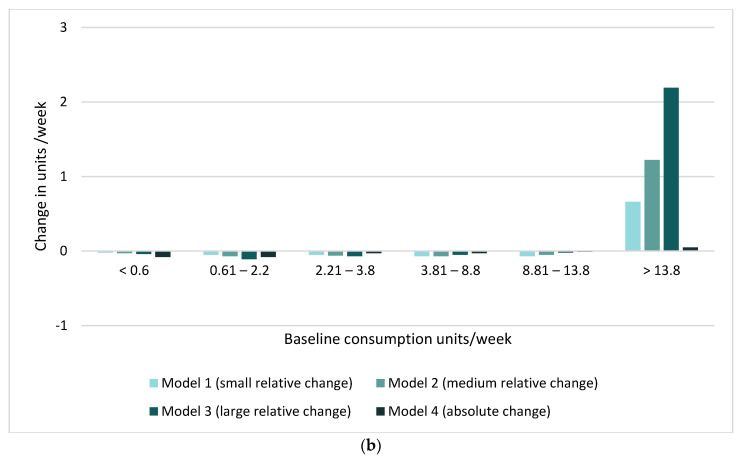
(**a**) Estimated change in the volume of consumption during the pandemic by baseline consumption level and four models for estimating change—ESAC survey. (**b**) Estimated change in volume of consumption during the pandemic by baseline consumption level and four models for estimating change—Opinion survey. Note 1: baseline consumption level categories correspond to intervals between the percentiles presented in Table 2, respectively.

We plotted change in consumption (estimated consumption during the pandemic minus baseline consumption) by level of baseline consumption and by model for estimating change (Figure 2). These showed that, irrespective of model, there was little change in consumption from baseline to during the pandemic in the lower and middle baseline consumption categories (i.e., mainly in the magnitude of 0–2 units/week in the ESAC survey and −0.1–0 units/week in the Opinion survey), whereas among those with the highest baseline consumption, there was a marked increase in consumption (i.e., in the magnitude of 7–13 units/week in the ESAC survey and 0.7–2.2 units/week in the Opinion survey).

Next, we explored whether the proportion of heavy drinkers, that is those exceeding various suggested limits for risk drinking, had increased from baseline to during the pandemic. With three suggested limits for risk drinking and four models for estimating change, we examined a total of 12 limit-model combinations for each survey. For the ESAC survey, the overall picture suggests that the proportion of heavy drinkers increased during the pandemic, the difference from baseline being statistically significant for most (10/12) limit-model combinations (Table 3). Moreover, the relative increase was higher with higher limits for risk drinking. For the Opinion survey, a similar pattern emerged, although due to the smaller sample size, the differences from baseline to during the pandemic were not statistically significant (Table 3).

## 4. Discussion

In two independent surveys, estimated mean alcohol consumption among adult drinkers in Norway changed very little in the first months of the COVID-19 pandemic, whereas the dispersion of the distribution increased. These estimates were based on assumptions of change being relative to that of baseline consumption. The magnitude and direction of estimated change in consumption were contingent on the baseline consumption level; while the vast majority of drinkers, on average, decreased their consumption slightly, the upper 5–10% of drinkers with the highest baseline consumption, increased their consumption substantially. In effect, the proportion of heavy drinkers increased from the baseline during the pandemic, despite small or no changes in average consumption.

Several survey studies have examined whether respondents had consumed more, the same, or less alcohol during the first months of the COVID-19 pandemic as compared to before the pandemic, demonstrating that a substantial proportion reported changing their alcohol use. Consistent with our findings, most studies found that a majority of those reporting a change, had reduced their drinking [10,14,15,18,21,31,32]. Moreover, our finding that an increase in drinking more often was reported when initial consumption was high, corroborates previous findings [9,20,24].

A novel contribution of this study is that we quantified reported changes in alcohol consumption. By doing so, we found an increase in consumption among those who drank the most initially, and thus, an increase in the proportion of heavy drinkers. This finding offers a more nuanced picture than the overall intuitive impression left from crude previous findings, which showed that less drinking responses outnumbered those of more drinking [10,14,15,18,21,31,32], and therefore, indicated a reduction in overall alcohol consumption during the first months of the pandemic [21].

Our finding that the estimated increase in consumption was particularly high among those with an initial high consumption corresponds to that reported by Mäkelä from Finland [33], although the situation in Finland was another; total consumption increased substantially from 1968 to 1969 due to increased alcohol availability.

Our study findings deviate to some extent from expectations about changes in alcohol consumption, based on the total consumption model [34,35] and the theory of collectivity of drinking [4]. While estimated consumption increased slightly or not at all during the pandemic, the underlying distribution changed in opposite directions; the lower percentiles decreased and the higher percentiles increased. In other words, consumer groups did not ‘move in concert’ (as formulated by Skog [4]), but they tended to polarize. Notably, this inconsistency should be regarded in context. Most studies of changes in alcohol consumption distribution have pertained to situations where the changes occurred in response to common stimuli and where collective changes could be expected, e.g., see [33]. During the COVID-19 pandemic, however, numerous societal measures as well as pandemic induced stress likely impacted differently on different consumer groups and different demographic groups. This may explain why we, under these circumstances, found a substantial increase in the prevalence of heavy drinkers, despite little or no change in total consumption. There are also other examples from the alcohol epidemiology of deviations from the total consumption model. One is from the abolition of alcohol rationing in Sweden, which—in short—implied that the prevalence of heavy drinkers increased dramatically despite little change in total consumption [36].

This study is based on data from a convenience sample and a web panel, and although data were weighted, we cannot rule out possible biases in the consumption distribution and reported changes in consumption during the pandemic in the Norwegian adult population. In survey research, heavy drinkers are typically under-represented, and alcohol use is under-reported by survey respondents [37]. Moreover, our survey failed to identify people who were abstinent before the pandemic but started drinking during the pandemic. Hence, the estimated increase in the dispersion of the distribution and the increase in the prevalence of heavy drinkers might in fact represent an underestimate. However, sales data and estimates of unrecorded consumption for Norway suggest a slight increase or no change in total alcohol consumption in the second quarter of 2020 (the first months of the pandemic) compared to the same period in 2019 [20], which corresponds quite well with our findings. Additionally, the ESAC survey seems to have reached heavier drinkers to a larger extent, since baseline consumption was slightly above the usual population average [22]. Our analyses are further based on theory-driven assumptions [4], and the results are only as robust as the assumptions. However, our research aimed to address the weaknesses of previous survey studies by quantifying the qualitative assessment of changes in consumption during the pandemic. This approach not only provided insights into the changes in alcohol consumption during the pandemic in Norway, but it may also serve as a useful tool for further studies of the pandemic’s impact on alcohol consumption.

Worries have been raised that the COVID-19 pandemic might have increased heavy drinking [38,39,40] and that the pandemic might result in more harmful consequences for those with an alcohol use disorder [41]. Our findings are the first to lend empirical support to these worries, suggesting that even with a very modest increase in total consumption, the proportion of heavy drinkers increased substantially. A substantial increase in the number of people with high risk of acute injuries and substantial risk of incident—or complications of existing—somatic or mental diseases [1,8], is indeed worrisome, and particularly so during a pandemic with high pressure on health services, as the services are burdened even more heavily. There is an urgent need to conduct further research into the possible effects of the pandemic on alcohol consumption distribution and risk drinking, as well as providing health support for those being at risk for increasing their drinking.

## 5. Conclusions

In conclusion, quantifications of reported changes in alcohol consumption during the pandemic suggest that the upper 5 to 10% of the drinkers increased their consumption and that the prevalence of heavy drinkers increased, despite little or no change in total consumption among drinkers.

## Figures and Tables

**Table 1:** Description of analytical survey samples by age and gender distribution.

		Weighted Survey Samples			
		ESAC Survey ^a^ (*n* = 15,267)		Opinion Survey ^b^ (*n* = 1195)	
		Per cent	*n*	Per cent	*n*
	Women	47.5	7245	49.6	592
Gender	Men	52.4	7998	50.4	603
	Other	0.2	24	NA	NA
	18–34 years	32.3	4932	27.6	330
Age groups	35–54 years	37.9	5791	35.5	424
	≥55 years	29.8	4544	36.9	441

^a^: See Kilian [30] for details regarding weighting procedures; ^b^: weighted by gender, age and geography; NA: not applicable.

**Table 2:** Mean, standard deviation and percentiles of alcohol units per week for baseline alcohol consumption and estimates for relative and absolute change. ESAC survey and Opinion survey (*n* = 15,267/n=1,195).

	Baseline Alcohol Consumption	Alcohol Consumption during the Pandemic			
		Assuming Relative Change			Assuming Absolute Change
		Model 1-Small	Model 2-Medium	Model 3-Large	Model 4
ESAC survey					
Mean	5.5	5.8	6.0	6.6	5.6
Standard deviation	8.6	10.4	11.7	14.8	9.5
Percentiles					
25	0.6	0.6	0.5	0.4	0.2
50	2.7	2.7	2.7	2.4	2.6
75	7.2	7.2	7.2	7.1	7.4
90	12.7	14.1	15.2	16.6	14.8
95	18.5	20.4	22.3	24.4	20.9
Opinion survey					
Mean	4.0	4.0	4.0	4.0	3.9
Standard deviation	5.5	5.7	5.9	6.2	5.6
Percentiles					
25	0.6	0.6	0.6	0.5	0.5
50	2.2	2.1	2.0	1.7	2.1
75	3.8	3.9	4.1	4.3	3.9
90	8.8	9.2	9.6	9.8	8.9
95	13.8	13.8	13.8	15.4	13.8

**Table 3:** Proportion of sample exceeding limits for risk drinking for baseline consumption and for estimated consumption during the pandemic by models—ESAC survey and Opinion survey. All values are given in percentage terms (%), *n* = 15,267/*n* = 1195.

		Alcohol Consumption during the Pandemic			
	Baseline Consumption	Assuming Relative Change			Assuming Absolute Change
		Model 1-Small	Model 2-Medium	Model 3-Large	Model 4
ESAC survey					
>14 units/week					
Total	9.5	10.2 *	10.4 **	11.4 ***	10.5 **
>21 units/week					
Total	4.6	4.9 ^ns^	5.2 **	6.7 ***	4.9 ^ns^
>28 units/week					
Total	1.8	3.3 ***	3.7 ***	4.2 ***	3.1 ***
Opinion survey ^a^					
>14 units/week					
Total	4.4	4.7	4.8	5.0	4.4
>21 units/week					
Total	2.2	2.2	2.8	2.8	2.2
>28 units/week					
Total	1.0	1.0	0.9	1.2	1.0

* *p* = 0.05, ** *p* < 0.01, *** *p* < 0.001 in Z-tests. Baseline consumption compared with estimated consumption during the pandemic. ^a^ none of the proportion differences between baseline consumption and consumption during the pandemic were statistically significant in the Opinion survey (i.e., *p* > 0.05).

## Data Availability

The dataset obtained in Survey 1 (ESAC) is available in the Figshare repository, https://doi.org/10.6084/m9.figshare.13580693.v1 (accessed on 15 January 2021).

## Supplementary Materials

The following are available online at https://www.mdpi.com/article/10.3390/ijerph18084231/s1, Table S1: overview of how qualitative responses to questions about changes in drinking frequency and usual quantity per occasion were translated into quantified measures of drinking frequency and usual quantity per occasion during the pandemic based on responses to questions on baseline consumption (AUDIT-C, Question 1 and Question 2) in four different models, for Survey 1 (ESAC).
