# Supplementary material for: Changes in Alcohol Consumption during the COVID-19 Pandemic—Small Change in Total Consumption, but Increase in Proportion of Heavy Drinkers
Source: Int J Environ Res Public Health. 2021 Apr 16;18(8):4231. doi: 10.3390/ijerph18084231 (PMC8073387; doi:10.3390/ijerph18084231)
Supplement: Supplementary file 1 [file ijerph-18-04231-s001.zip › ijerph-1149732-supplementary.pdf]

Supplementary Table S1. Overview of how qualitative responses to questions about changes in drinking frequency and usual quantity per occasion were translated into quantified measures of drinking frequency and usual quantity per occasion during the pandemic based on responses to questions on baseline consumption (AUDIT-C, question 1 and question 2) in four different models, for Survey 1 (ESAC). Models 1, 2 and 3 assume change relative to the baseline level, whereas Model 4 assumes an absolute change, irrespective of baseline level.

		Models assuming relative change															Model assuming absolute change				
		Model 1 - small					Model 2 - medium					Model 3 - large					Model 4				
		Much less	Slightly less	No change	A little more	Much more	Much less	A little less	No change	A little more	Much more	Much less	A little less	No change	A little more	Much more	Much less	A little less	No change	A little more	Much more
**AUDIT-C question 1**																					
Quantified change		-23%	-5%	0	+5%	+30%	-33%	-9%	0	+10%	+50%	-50%	-13%	0	+15%	+100%	-50	- 10	0	+ 10	+50
Monthly or less	= 6[a]	4.6	5.7	6	6.3	7.8	4	5.5	6	6.6	9	3	5.2	6	6.9	12	0	0	6	16	56
2-4 times a month	=40[a]	30.8	38	40	42	52	26	36	40	44	60	20	35	40	46	80	0	30	40	50	90
2-3 times a week	=120[a]	92.4	114	120	126	156	79	109	120	132	180	60	104	120	138	240	70	110	120	130	170
4 + times a week	=250[a]	192,5	237.5	250	262.5	345	165	228	250	275	365	125	218	250	288	365	200	240	250	260	300
**AUDIT-C question 2**																					
Quantified change		-23%	-5%	0	+5%	+30%	-33%	-9%	0	+10%	+50%	-50%	-13%	0	+15%	+100%	-1.0	- 0.1	0	+ 0.1	+1.0
1 or 2	=1.5[b]	1.2	1.4	1.5	1.6	2.0	1.0	1.4	1.5	1.7	2.3	0.7	1.3	1.5	1.7	3.0	0.5	1.4	1.5	1.6	2.5
3 or 4	=3.5[b]	2.7	3.3	3.5	3.7	4.6	2.3	3.2	3.5	3.9	5.3	1.8	3.0	3.5	4.0	7.0	1.5	3.4	3.5	3.6	4.5
5 or 6	=5.5[b]	4.2	5.2	5.5	5.8	7.2	3.6	5.0	5.5	6.1	8.3	2.8	4.8	5.5	6.3	11.0	4.5	5.4	5.5	5.6	6.5
7-9	=8.0[b]	6.2	7.6	8.0	8.4	10.4	5.3	7.3	8.0	8.8	12.0	4.0	7.0	8.0	9.2	16.0	7.0	7.9	8.0	8.1	9.0
10 or more	=12.0[b]	9.2	11.4	12.0	12.6	15.6	7.9	10.9	12.0	13.2	18.0	6.0	10.4	12.0	13.8	24.0	11.0	11.9	12.0	12.1	13.0

[a] Given value for number of drinking occasions in the past 12 months; [b] Given value for usual number of alcohol units per drinking occasion
